# Supplementary material for: Study protocol for a pilot clinical trial to understand neural mechanisms of response to a psychological treatment for pain and anxiety in pediatric functional abdominal pain disorders (FAPD)
Source: PLoS One. 2024 Mar 18;19(3):e0299170. doi: 10.1371/journal.pone.0299170 (PMC10947640; doi:10.1371/journal.pone.0299170)
Supplement: S1 File — (DOCX) [file pone.0299170.s002.docx]

**Michigan State University Human Research Protection Program**

| - Complete this template for new exempt, expedited, or full board studies.   - Complete Section I for ALL studies (exempt, expedited, full board)   - Complete Section II ONLY if your study does not qualify for exemption and requires an expedited or full board review. Contact the IRB office if you have any questions. - CLICK™ IRB:   - Include the template with a New Study Submission.   - Upload the completed template to the Basic Information SmartForm page, Question 10.   - When uploading documents to Click (e.g. consent documents, instrument), provide distinct file names. - See the Click Quick Guides and the HRPP Manual for more information, available at hrpp.msu.edu | | | | | | | |
| --- | --- | --- | --- | --- | --- | --- | --- |
|  | | | | | | | |
| **Study Title:** | | | | | Using fMRI to understand response to an integrative treatment for pain and anxiety in pediatric functional abdominal pain disorders (FAPD). | | |
| **Click Study ID (if known):** | | | | | STUDY00003419 | | |
| **Sponsor (if applicable):** | | | | |  | | |
| **Sponsor ID (if applicable):** | | | | |  | | |
|  | | | | | | | |
| **Section I. IRB Protocol for All Studies**  Section I is completed for ***all studies*** and includes questions to determine whether the study qualifies for exemption. Section II is only completed if the study does not qualify for exemption. | | | | | | | |
|  | | | | | | | |
| **1.** | **Hypothesis / Objective / Goals / Aims.** | | | | | | |
|  | Briefly describe the study’s hypothesis / objectives / goals / aims. | | | | | | |
|  | Aim 1. Left AMY-PFC functional connectivity will be significantly diminished (i.e., evidence of decreased hyperconnectivity) during the water load symptom provocation task (WL-SPT) post ADAPT vs. waitlist.  Aim 2. Brain activations associated with cognitive (PFC), affective (pgACC, AMY), and visceral afferent (INS, thalamus, aMCC, S1 & S2) pain will significantly be more diminished after ADAPT vs. waitlist.  Exploratory Aim. Changes in functional connectivity and brain activations following ADAPT will correspond to reductions in pain (intensity and unpleasantness) and anxiety ratings. | | | | | | |
| **2.** | **Subject Population.** | | | | | | |
| **2A.** | Study purposefully includes the following subject population(s) (select all that apply): | | | | | | |
|  | Cognitively impaired adults  Minors (children) (view information about the definition of a child)  Minors who are wards of the state  Pregnant women, fetuses, or neonates  Prisoners  Students | | | | | | |
| **2B.** | Study involves (select all that apply): | | | | | | |
|  | Funding, support, or other requirement to comply with U.S. Department of Justice regulations  Incomplete disclosure or attempted deception of subjects | | | | | | |
|  | *CLICK IRB: Upload the debriefing script, document, etc. to the Consent Forms and Recruitment Materials SmartForm page, Question 1.* | | | | | | |
| **3.** | **Estimated Study Duration.** | | | | | | |
|  | Provide the time estimated to complete all human subject research, including analysis of the subjects’ identifiable private information. | | | | | | |
|  | As part of ongoing data collection, participants will be recruited from GI or behavioral medicine clinics, or be recruited from a previous study of youth with FAPD, at Spectrum/DeVos Children's Hospital and enrolled in the study during that visit (immediately). They will then come in for the baseline assessment, which will occur approximately one week after enrollment (though up to six weeks after enrollment will be allowed to account for MRI scanner availability). Following that visit, they will then be randomized to either the ADAPT treatment group or a waitlist control group (each six weeks in duration) and will be informed of group assignment within a week of their baseline assessment, and will begin ADAPT/waitlist approximately one week after group assignment (with up to three weeks allowed to begin ADAPT). Upon completion of ADAPT or waitlist control, participant outcomes will be reassessed at the post assessment visits (approximately eight weeks after the baseline). After completing the post assessment visit, participants in the waitlist control group will be given the opportunity to complete ADAPT.  Participants randomized to ADAPT will be actively involved in the study for a ~10 week period (from screening to post assessment). Those who are randomized to the waitlist control and then opt to receive ADAPT afterwards will be involved for ~16 weeks.  It is anticipated that we will be actively enrolling participants for 1.5 years. After study completion, three additional months will be allotted for data analysis.  We note that data will also be included from prior site (Cincinnati Children's) where data was collected as part of an IRB-approved investigation. No data collection is currently ongoing at Cincinnati Children's. | | | | | | |
| **4.** | **Reasonably Foreseeable Risks.** | | | | | | |
| **4A.** | There are (select one of the following): | | | | | | |
|  | No reasonably foreseeable risks to subjects  Reasonably foreseeable risks to subjects | | | | | | |
| **4B.** | Explain the selection. *If you selected that there are reasonably foreseeable risks to subjects, describe the risks, considering physical, psychological, social, legal and economic risks.* | | | | | | |
|  | Participant safety will be monitored once an individual is enrolled in the study. Potential risks and adverse events are listed below:  • Emotional Distress. Given the risk of elevated anxiety and mood problems in individuals with functional abdominal pain disorders (FAPD), some responses on these measures may reveal anxiety, depressive affect, and/or suicidal thoughts. Youth may also find some questions embarrassing or uncomfortable to talk about. Although ADAPT online modules do not specifically elicit responses that may reveal depression/suicidal ideation, such symptoms (anxiety and mood) may arise during ADAPT.  • fMRI related Risks. fMRI has been approved for routine research and clinical applications and does not pose any known risk to participants. There are no known risks from exposure to the magnetic fields and radio waves used during fMRI data collection. However, it is not assured that harmful effects will not be recognized in the future. It is unknown if/how the MRI machine effects pregnancy. A known risk is that strong magnetic fields attract iron or steel metal objects, thus posing a safety risk. In addition, it is possible that participants may feel uncomfortable or confined once inside the imaging machine.  • Time Commitment and Fatigue. The assessment visits will require approximately a 2 hour time commitment (clinical measures/fMRI visit), and children will need to sit still for the duration of scans (approximately 1-hour) which may cause slight discomfort  • The water loading task is a non-invasive and validated procedure for induction of abdominal discomfort in youth with FAPD. The procedure was validated by Walker and colleagues (2006). Children are, by design, likely to experience abdominal discomfort during the task. The procedure produces symptoms similar to but less intense than those naturally experienced by children with FAPD. There is a small risk of vomiting if the child consumes water beyound the point of feeling completely full. | | | | | | |
| **4C.** | If you selected that there are reasonably foreseeable risks, describe the procedures for protecting against or minimizing potential risks and provide an assessment of their likely effectiveness. | | | | | | |
|  | Emotional Distress  To help reduce risk, participants will complete a standardized depressive symptoms screener where suicidal ideation will be directly queried. This will occur prior to engaging in the fMRI portion of the study Participants with high levels of depression (cut-score >= 20) and/or active suicidal ideation will be referred for mental health care and will not be eligible for the study. Responses will be montified and particpants will receive a follow up evaluation if such symptoms are endorsed. In the event that a participant reveals severe depressive symptoms or suicidal ideation, the following steps will be taken: a risk assessment will occur under the supervision of Dr. Barber-Garcia (licensed clinical psychologist). A professional and confidential risk assessment, including detailed information about suicidal ideation, intent and/or plans, access to means to hurt themselves, major stresses, availability of social supports, access to treatment, and plans for safety will be discussed in detail with the participant and their parent. The assessment will be conducted by a study interventionist with clinical oversight from Dr. Barber Garcia. A referral to the ER and/or a referral to appropriate clinical care will be made. If the family refuses to follow through on the aforementioned recommendations, we will contact the appropriate authorities as warranted to ensure the safety of our participants. All actions will be documented.  During assessment and/or treatment procedures, participants may reveal experiences of abuse to the assessor or study therapist. In the event that the project staff becomes aware of suspected or actual abuse or neglect, Dr. Barber Garcia will be notified. A report will be immediately filed with the appropriate state agency when necessary. The informed consent/assent procedures specify that confidentiality will be breached if research staff learn that a minor is the victim or suspected victim of abuse or neglect.    For ADAPT participants, a safety assessment will occur via phone if indicated. Of note ADAPT is an evidence-based intervention and has not been found to be associated with any adverse effects. All participants will receive their medical care as usual during their participation in this study.  fMRI related Risks  Prior to participation in the fMRI scans, participants will be given questionnaires to determine if they if they are eligible to complete the fMRI procedure. If they have metal objects in their bodies, they will be excluded from participating in the study. In addition, any removable metal (e.g., glasses, watch, clothes with zippers) on the day of imaging will be removed before the participant enters the fMRI rooms. Based on the extent literature, there are no known risks to pregnant individuals or the fetus undergoing standard fMRI procedures in an MRI scanner.  Ray JG, Vermeulen MJ, Bharatha A, Montanera WJ, Park AL. Association between MRI exposure during pregnancy and fetal and childhood outcomes. JAMA. 2016;316(9):952-961.  Lum M, Tsiouris AJ. MRI safety considerationd during pregnancy. Clin Imaging. 2020;62:69-75.  However, there may be risks not yet known. Therefore female participants will be asked whether or not they are currently pregnant or suspect to be pregnant. Participants who are pregnant or suspect pregnancy will be excluded from participating in the study.  During the imaging portion of the study, any participant who experiences discomfort or exhibits distress will be monitored visually and via microphone to ensure they are tolerating the procedure. If participants express a desire to leave the machine, either temporarily or permanently at any point, they will be removed immediately.  An on-call psychologist will be available during MRI visits for psychological distress.  Time Commitment and Fatigue  Assessors will be trained to assess fatigue, and will give participants a 5-10 minute break if needed. For ADAPT sessions, there should be minimal discomfort due to the shorter length of these sessions (60 minutes or less).  Participants will be informed of their right to refuse to participate in any part of the data collection and will be given the phone numbers of the Principal Investigator as well as the Institutional Review Board of MSU in the event that they desire further information or would like to issue a formal complaint.    Water Loading Task  As noted by the authors, “This level of discomfort was acceptable to children and their parents” (Walker et al., 2006, p. 710). It will be explained to families that participation is completely voluntary and that they may drop out of the study at any time, for any reason, and that this will not affect the child’s medical care. During the water load period, children will be asked to rate their fullness at 5-minute intervals – to make sure they do not push themselves to consume water beyond the point of perceived fullness. One child (out of 230) in Walker’s original study vomited following water ingestion. “During debriefing, the child reported that he had pushed himself to drink water beyond the point of feeling full. In subsequent administrations of the water load, children were cautioned that vomiting was a possibility if they continued to consume water beyond the point of feeling completely full.” (Walker et al., 2006; p. 707). We will caution children similarly in our study.  Another unlikely concern is exceptionally rare occurrence of water toxicity. To eliminate this risk, a daily fluid maintenance formula will be used based on their weight to determine the maximum fluid value for each child. The amount of water will be capped at that value (up to 1.5 L). Further, allowing a specific time frame (up to 15 minutes) creates conditions to make water toxicity impossible. Study staff will be on hand to ensure the participant stops drinking water after a complete sensation of fullness. In addition, participants will be instructed that they are free to terminate the task at any time. | | | | | | |
| **5.** | **Conflict of Interest.** | | | | | | |
|  | Do any investigators or research staff have a financial interest related to the research that has not otherwise been disclosed elsewhere in this submission? | | | | | | No  Yes |
| **6.** | **Exemption Criteria.** | | | | | Not Applicable | |
|  | A study may qualify for exemption when the only involvement of human subjects will be in one or more of the following categories (please view full exemption category / description here: https://hrpp.msu.edu/help/required/exempt-categories.html). **(*If the study does not qualify for the exemption criteria, do not complete this question and proceed to Section II.)*** | | | | | | |
| **6A.** | Exemption Categories. | | | | | | |
|  | **6A1.** | Select the category(ies) applicable to the study if the only involvement of human subjects in this study will be in one or more of the categories. Studies involving prisoners cannot be exempt UNLESS the research is aimed at involving a broader subject population that only incidentally includes prisoners *If your study is subject to U.S. Department of Justice requirements, do not complete this section; complete 6A2 below.* | | | | | |
|  |  | ***Exempt 1.*** Research conducted in established or commonly accepted educational settings, involving normal educational practices that are not likely to adversely impact students' opportunity to learn required educational content or the assessment of educators who provide instruction. ***IF YOU SELECTED THIS CATEGORY, EXPLAIN WHY THE RESEARCH WILL NOT LIKELY ADVERSELY IMPACT STUDENTS’ OPPORTUNITY TO LEARN REQUIRED EDUCATIONAL CONTENT OR THE ASSESSEMENT OF EDUCATORS WHO PROVIDE INSTRUCTION.*** | | | | | |
|  |  |  | | | | | |
|  |  | ***Exempt 2.*** Research that only includes interactions involving educational tests (cognitive, diagnostic, aptitude, achievement), survey procedures, interview procedures, or observation of public behavior. ***IF YOU SELECTED THIS CATEGORY, SELECT THE APPROPRIATE OPTION(S) BELOW.*** | | | | | |
|  |  |  | (i) Information obtained is recorded by investigator in manner that identity of subjects cannot readily be ascertained, directly or through identifiers linked to subjects | | | | |
|  |  |  | (ii) Any disclosure of subjects' responses outside research would not reasonably place subjects at risk of criminal or civil liability or be damaging to subjects' financial standing, employability, educational advancement, or reputation. | | | | |
|  |  |  | (iii) **LIMITED IRB REVIEW REQUIRED**. Information obtained is recorded by investigator in manner that identity of subjects can readily be ascertained, directly or through identifiers linked to subjects, and responses could reasonable place subjects at risk of criminal or civil liability or be damaging to the subjects' financial standing, employability, educational advancement, or reputation ***(LIMITED IRB REVIEW IS REQUIRED; YOU MUST ALSO COMPLETE QUESTION 6E TO DESCRIBE PRIVACY AND CONFIDENTIALITY SAFEGUARDS.)*** | | | | |
|  |  | ***Exempt 3.*** Research involving benign behavioral interventions in conjunction with the collection of information from an adult subject through verbal or written responses (including data entry) or audiovisual recording if the subject prospectively agrees to the intervention and information collection. ***IF YOU SELECTED THIS CATEGORY, SELECT THE APPROPRIATE OPTION(S) BELOW.*** | | | | | |
|  |  |  | (i) Information obtained is recorded by investigator in manner that identity of subjects cannot readily be ascertained, directly or through identifiers linked to subjects. | | | | |
|  |  |  | (ii) Any disclosure of subjects' responses outside research would not reasonably place subjects at risk of criminal or civil liability or be damaging to subjects' financial standing, employability, educational advancement, or reputation | | | | |
|  |  |  | (iii) **LIMITED IRB REVIEW REQUIRED**. Information obtained is recorded by investigator in manner that identity of subjects can readily be ascertained, directly or through identifiers linked to subjects, and responses could reasonable place subjects at risk of criminal or civil liability or be damaging to subjects' financial standing, employability, educational advancement, or reputation ***(LIMITED IRB REVIEW IS REQUIRED; YOU MUST ALSO COMPLETE QUESTIONS 6E TO DESCRIBE PRIVACY AND CONFIDENTIALITY SAFEGUARDS.)*** | | | | |
|  |  | ***Exempt 4.*** Secondary research uses of identifiable private information or identifiable biospecimens. ***IF YOU SELECTED THIS CATEGORY, SELECT THE APPROPRIATE OPTION(S) BELOW.*** | | | | | |
|  |  |  | Identifiable private information or identifiable biospecimens are publicly available. | | | | |
|  |  |  | Information, which may include information about biospecimens, is recorded by the investigator in such a manner that the identity of the human subjects cannot readily be ascertained directly or through identifiers linked to the subjects, the investigator does not contact the subjects, and the investigator will not re-identify subjects. ***IF YOU SELECTED THIS CATEGORY, CONFIRM THE FOLLOWING:*** | | | | |
|  |  |  |  | Investigator and research team will not contact the subjects  Investigator and research team will not re-identify the subjects | | | |
|  |  |  | The research involves only information collection and analysis involving the investigator's use of identifiable health information when that use is regulated under the Health Insurance Portability and Accountability Act (HIPAA) 45 CFR parts 160 and 164. | | | | |
|  |  |  | The research is conducted by, or on behalf of, a Federal department or agency using government-generated or government-collected information obtained for nonresearch activities, if the research generates identifiable private information that is or will be maintained on information technology that is subject to and in compliance with specific federal privacy standards. | | | | |
|  |  | ***Exempt 5.*** Federal demonstration projects. | | | | | |
|  |  | ***Exempt 6.*** Taste and food quality evaluation and consumer acceptance studies. | | | | | |
|  |  | ***Exempt 97***. ONLY applicable to research NOT FUNDED by a federal department or agency: Research involving the study of previously collected identifiable data (please view additional exclusions before selecting this category). | | | | | |
|  |  | *By checking the boxes below, you are confirming that the study will not include any of the following exclusions for the study’s duration:*  Federal funding or federal training grants  FDA regulated  Sponsor or other contractual restrictions  Clinical interventions (including clinical behavioral interventions)  Receipt of an NIH issued certificate of confidentiality to protect identifiable research data  Multi-site collaborative research study where another institution plans to rely or is relying upon MSU’s IRB review | | | | | |
|  |  | ***Exempt 98.*** ONLY applicable to research NOT FUNDED by a federal department or agency: Prospective data collection with adults through verbal or written responses involving a benign intervention (please view additional exclusions before selecting this category). | | | | | |
|  |  | *By checking the boxes below, you are confirming that the study will not include any of the following exclusions for the study’s duration:*  Federal funding or federal training grants  FDA regulated  Sponsor or other contractual restrictions  Clinical interventions (including clinical behavioral interventions)  Receipt of an NIH issued certificate of confidentiality to protect identifiable research data  Multi-site collaborative research study where another institution plans to rely or is relying upon MSU’s IRB review  Children as research subjects | | | | | |
|  | **6A2.** | **DEPARTMENT OF JUSTICE Exemption Categories*.*** Complete this section ONLY if the research is subject to Department of Justice requirements. | | | | | |
|  | **6A2i.** | Select the category(ies) applicable to the study if the only involvement of human subjects in this study will be in one or more of the categories. Studies involving prisoners cannot be exempt. | | | | | |
|  |  | ***Exempt 1.*** Research conducted in established or commonly accepted educational settings, involving normal educational practices. | | | | | |
|  |  | ***Exempt 2.*** Educational tests, survey procedures, interview procedures, observation of public behavior unless data is recorded in a manner such that subjects are identifiable and the responses could reasonably place the subjects at risk of criminal or civil liability or be damaging to the subjects’ financial standing, employability, or reputation (research cannot involve children, except for educational tests or observation of public behavior where the investigator does not interact with the child). | | | | | |
|  |  | ***Exempt 3.*** Educational tests, survey procedures, interview procedures, or observation of public behavior not otherwise exempt that involves public officials or federal statute. | | | | | |
|  |  | ***Exempt 4.*** Collection or study of existing data, documents, records, pathological specimens, or diagnostic specimens if publicly available or information is recorded by investigator in a manner that subjects cannot be identified. | | | | | |
|  |  | ***Exempt 5.*** Federal demonstration projects. | | | | | |
|  |  | ***Exempt 6.*** Taste and food quality evaluation and consumer acceptance studies. | | | | | |
|  | **6A2ii.** | Explain why the study presents minimal risk to subjects. | | | | | |
|  |  | Overall, the study does not significantly increase the participants’ risk of harm beyond those risks that are inherent in ordinary daily living. All study procedures can be terminated immediately. In addition, all participants will be able to receive a potentially beneficial intervention (ADAPT) to manage pain and anxiety. Information obtained from this study will be valuable for refining behavioral interventions for the treatment of youth with chronic pain and comorbid anxiety. | | | | | |
| **6B.** | By checking the boxes below, you are confirming that the following are true and will remain true for the study’s duration: | | | | | | |
|  | Selection of subjects is equitable (considering the purposes of the research, setting in which research will be conducted, any vulnerable populations).  If there is recording of identifiable information, there are adequate provisions to maintain the confidentiality of the data.  There are adequate provisions to maintain the privacy interests of subjects.  Safeguards are or will be put in place to protect against any coercion or undue influence if you or members of your study team are or may be associated with the subjects at any point in the study (e.g. students, employees, colleagues, patients). | | | | | | |
| **6C.** | Consent | | | | | | |
| **6Ci.** | There will be a consent process for the study’s duration that will disclose information such as that the activity involves research, a description of the procedures, that participation is voluntary and withdrawal is without penalty, and the name and contact information for the researcher (select appropriate option below): | | | | | | |
|  | For All Subjects  For Some Subjects  For None of the Subjects (consent will not be obtained)  *CLICK IRB: Upload the consent document to the Consent Forms and Recruitment Materials SmartForm page.* | | | | | | |
| **6Cii.** | Please explain your selection. | | | | | | |
|  | A signed assent/consent/peromisison form will be obtained from each participant and their legal guardian (e.g., person with power of attorney) before completing any study activities. | | | | | | |
| **6D.** | Please acknowledge that you may not begin the research at non-MSU institutions (regardless of engagement), until you receive the appropriate approvals/permissions from the sites (e.g. IRB review/exempt determination from non-MSU sites, data use or research agreements, other regulatory approvals). An MSU exempt determination does not provide approval/permission for a non-MSU site, including sites with reliance agreements with MSU. Please note that non-MSU sites may have requirements that differ from MSU for exempt research. Note that this also applies to sites added after the MSU exempt determination. | | | | | | |
|  | Acknowledged | | | | | | |
| **6E.** | **LIMITED IRB REVIEW.** If the exemption(s) require limited IRB review (if you selected Exemption 2(iii) or 3(i)(C) in Question 6A), complete questions 1 and 2 to describe privacy and confidentiality. | | | | | | |
| **6E1.** | **Privacy of Subjects.** | | | | | | |
|  | How will subjects’ privacy be protected? Consider the number of individuals interacting with the subject or subject’s records, location of consent process and study, presence of individuals not associated with the study, sensitivity of the research. | | | | | | |
|  | There is a minimal risk that the data collected for each participant may be viewed by individuals outside the research team. To minimize risk to confidentiality, every effort will be made to ensure that research data are kept confidential and stored so that data cannot be accessed by individuals who are not part of the research team. Unique identification numbers will be assigned to participants, and all case report forms will be coded with this number rather than a name. A password-protected master list linking the identification number to participant names will be stored on a secure computer separate from the study data. Access to the master list will be limited to key study personnel. Upon study completion, all study materials and participants’ personal information will be destroyed. Locked filing space within the Secchia Center research laboratory will be identified and used exclusively for the purposes of this study.  All consent forms, contact information and identifying data will be stored in a secure location within the Secchia Center research lab or on a secure computer. The subject codebook will be stored separately in a password-protected document. Regarding the use of online measures and web modules, material development will be conducted in accordance with MSU policies. Hardware for this study will be provided and maintained by MSU Informatics, which maintains a secure web server for supporting projects that potentially contain protected health information (PHI) and are subsequently subject to compliance with federal and state regulations regarding data of this type.  Brain image(s) will be provided to the participating family upon request. Images will be transferred via encrypted email. | | | | | | |
| **6E2.** | **Confidentiality of Data.** | | | | | | |
| **6E2i.** | Select the appropriate option: | | | | | | |
|  | Identifying or coded information will not be stored with the information and/or biospecimen(s)  Identifying or coded information will be stored with the information and/or biospecimen(s) | | | | | | |
| **6E2ii.** | Please explain your selection. If you are storing identifying or coded information with the information and/or biospecimen(s), explain why identifiable or coded information and/or biospecimen(s) needs to be maintained and how long it will be necessary to maintain it. | | | | | | |
|  | Identifiable information may be stored on a secure REDCap database which will also contain the participant's unique idenitfication number. However, this identifiable information will be stripped for data analysis. | | | | | | |
| **6E2iii.** | Describe the procedures and safeguards you will use to secure the information and/or biospecimen(s), including during transport of information and/or biospecimen(s). | | | | | | |
|  | All data will be identified with ID numbers and kept in locked files in a space in the Secchia Center research laboratory or on a secure computer that is designated specifically for the purposes of this project. All de-identified data (with the exception of fMRI data) will be saved into REDCap, a password-protected database. Data output will be stored on a network devoted solely to the research activities of MSU Department of Family Medicine (DFM). Electronic data stored on the MSU network is backed up regularly. The High-Performance Computing Center (HPCC) will be used for checking, storing, and analyzing fMRI data. The HPCC is maintained by MSU's Institutte for Cyber-Enabled Research (ICER). The fMRI data will also be stored on a secure server, backed up nightly, and will only be accessible to study staff. | | | | | | |
|  |  | | | | | | |

| **Other Click IRB Documents to Upload As Appropriate**  **(Applicable to All Studies)** |
| --- |
| - *Upload this completed protocol to the Basic Information SmartForm page, Question 10.* - *Upload any funding materials not accessible in Kuali Coeus in the Supporting Documents SmartForm page.* - *Upload the HRP-537 - Template - Use of Protected Health Information Application to the MSU Additional Study Information SmartForm page.* - *Upload the HRP-538 - Template - MSU Authorization to Use or Disclose Health Information for Researchers to the MSU Additional Study Information SmartForm page.* |
|  |
| **IF THE STUDY MAY QUALIFY FOR AN EXEMPTION**  **(INCLUDING THOSE THAT MAY REQUIRE LIMITED IRB REVIEW),**  **STOP HERE AND DO NOT COMPLETE SECTION II.**  **CONTINUE ONLY IF THE STUDY**  **DOES NOT QUALIFY FOR AN EXEMPTION.**  **COMPLETE QUESTIONS 7-23 FOR**  **AN EXPEDITED OR FULL BOARD STUDY.** |

| **Section II. Additional Questions for an Expedited or Full Board Study**  Not all questions or sections are applicable to every study. If the question or section is not applicable, check the “Not Applicable” box. All other questions are required. | | | | | | | | |
| --- | --- | --- | --- | --- | --- | --- | --- | --- |
| **7.** | **Expedited Categories.** | | | | | | | |
| **7A.** | Please select the Expedited category(ies) and sub-categories as applicable to the study if the only involvement of human subjects in this study will be in one or more of the categories. If the study involves more than minimal risk or none apply, select “The study involves more than minimal risk OR none of the expedited category(ies) apply.” | | | | | | | |
|  |  | ***The study involves more than minimal risk OR none of the expedited categories apply. IF THIS OPTION IS SELECTED, DO NOT SELECT ANY OF THE EXPEDITED CATEGORY(IES).*** | | | | | | |
|  |  | ***Expedited 1*.** Clinical studies of drugs and medical devices only when condition (a) or (b) is met. ***IF YOU SELECTED THIS CATEGORY, SELECT THE APPROPRIATE OPTION(S) BELOW.*** | | | | | | |
|  |  |  | (a) Research on drugs for which an investigational new drug application (21 CFR Part 312) is not required. (Note: Research on marketed drugs that significantly increases the risks or decreases the acceptability of the risks associated with the use of the product is not eligible for expedited review.) | | | | | |
|  |  |  | (b) Research on medical devices for which (i) an investigational device exemption application (21 CFR Part 812) is not required; or (ii) the medical device is cleared/approved for marketing and the medical device is being used in accordance with its cleared/approved labeling. | | | | | |
|  |  | ***Expedited 2*.** Collection of blood samples by finger stick, heel stick, ear stick, or venipuncture. ***IF YOU SELECTED THIS CATEGORY, SELECT THE APPROPRIATE OPTION(S) BELOW.*** | | | | | | |
|  |  |  | (a) from healthy, nonpregnant adults who weigh at least 110 pounds. For these subjects, the amounts drawn may not exceed 550 ml in an 8 week period and collection may not occur more frequently than 2 times per week; or | | | | | |
|  |  |  | (b) from other adults and children [2], considering the age, weight, and health of the subjects, the collection procedure, the amount of blood to be collected, and the frequency with which it will be collected. For these subjects, the amount drawn may not exceed the lesser of 50 ml or 3 ml per kg in an 8 week period and collection may not occur more frequently than 2 times per week | | | | | |
|  |  | ***Expedited 3***. Prospective collection of biological specimens for research purposes by noninvasive means. | | | | | | |
|  |  | ***Expedited 4***. Collection of data through noninvasive procedures (not involving general anesthesia or sedation) routinely employed in clinical practice, excluding procedures involving x-rays or microwaves. Where medical devices are employed, they must be cleared/approved for marketing. (Studies intended to evaluate the safety and effectiveness of the medical device are not generally eligible for expedited review, including studies of cleared medical devices for new indications.) | | | | | | |
|  |  | ***Expedited 5***. Research involving materials (data, documents, records, or specimens) that have been collected, or will be collected solely for nonresearch purposes (such as medical treatment or diagnosis). | | | | | | |
|  |  | ***Expedited 6***. Collection of data from voice, video, digital, or image recordings made for research purposes. | | | | | | |
|  |  | ***Expedited 7***. Research on individual or group characteristics or behavior (including, but not limited to, research on perception, cognition, motivation, identity, language, communication, cultural beliefs or practices, and social behavior) or research employing survey, interview, oral history, focus group, program evaluation, human factors evaluation, or quality assurance methodologies. | | | | | | |
| **7B.** | **For Studies Regulated by the U.S. Food and Drug Administration or the U.S. Department of Justice**. If you selected an expedited category, explain why the study presents minimal risk to subjects. | | | | | | | |
|  |  | | | | | | | |
| **8.** | **More than Minimal Risk Research.** *Complete the following question if you selected “The study involves more than minimal risk OR none of the expedited categories apply” in Question 7A (Expedited Categories).* | | | | | | | |
| **8A.** | Describe the relevant prior experience and gaps in current knowledge, relevant preliminary data, if any, and the scholarly background for, and significance of, the research based on existing literature and how it will add to existing knowledge. | | | | | | | |
|  |  | | | | | | | |
| **8B.** | Sample Size. | | | | | | | |
| **8Bi.** | Total number of subjects who will be approached (including screen failures, controls and subject withdrawals) to reach enrollment numbers for the lifetime of the study at this investigator’s sites. | | | | | | | |
|  |  | | | | | | | |
| **8Bii.** | Total number of subjects who will be enrolled in the study at this investigator’s site. | | | | | | | |
|  |  | | | | | | | |
| **8Biii.** | Describe the statistical justification or rationale for the proposed sample size. Considerations for sample size may include the acceptable level of significance, power of the study, expected effect size, underlying event rate in the population, standard deviation in the population, saturation of themes, and/or have a theoretical basis. | | | | | | | |
|  |  | | | | | | | |
| **9.** | **Minimal Risk Research.** *Complete the following question if you selected an expedited category in Question 7A.* | | | | | | | |
| **9A.** | Briefly describe the background for conducting the research. (1-2 sentences) | | | | | | | |
|  | The Aim to Decrease Pain and Anxiety Treatment (ADAPT) is a cognitive behavioral approach developed by the PI (Cunningham) to manage pain and anxiety for youth with FAPD and comorbid anxiety. ADAPT may be effective, but it is unknown how the intervention works. Thus, the current study seeks to understand the neural mechanisms underlying response to ADAPT. | | | | | | | |
| **9B.** | Sample Size. | | | | | | | |
| **9Bi.** | Provide an estimated sample size for the lifetime of the study at this investigator’s sites. | | | | | | | |
|  | The study population is male and female youth between the ages of 11-16 diagnosed with functional abdominal pain disorders (FAPD) and clinically significant anxiety. We plan to approach approximately 124 participants, ages 11-16 years. Of those, we expect 75% will agree to participate (n=93) based on our pilot fMRI study. Of those, we expect approximately 65% will qualify. Thus, we anticipate recruiting n=60 to complete a baseline assessment. Based on our previous RCT, we expect 85% of those recruited in clinic will complete the baseline assessment (n = 50) and the majority will be retained in the study (90%, n=45). Based on our fMRI pilot study, we expect to lose approximately 10% of participant data to movement artifacts yielding n=40 with usable data. The minimum number of total completed participants allowed will be 34 and the maximum will be 50. We aim to have between 17 – 25 participants in each arm for a total N of 34 - 50 participants in the study. Gender and age will be used as blocking variables in randomization. | | | | | | | |
| **9Bii.** | Describe the basis for that estimate. | | | | | | | |
|  | We’ve used the following tool (http://neuropowertools.org/neuropower/neuropowerinput/) to conduct sample size calculations for the fMRI portion of the study. Power calculations were based on prior studies of functional connectivity data 1) pre and post psychological therapy for pediatric pain (migraine), and 2) comparing individuals with pediatric pain (migraine) to healthy controls. While these groups are not synonymous with those proposed in the current study (which aims to compare youth with FAPD who have received psychological therapy for pain and anxiety to those in a waitlist control condition), this preliminary data yields meaningful information by which we can estimate the power required for the proposed investigation. The power calculations and sample size requirements are detailed below:  For within group changes, we relied on the pre/post data following a psychological therapy for pediatric pain (migraine). Here we found that a total sample size of 34 would be required for power of .8 and p < 0.05. For between group changes, we utilized data comparing youth with chronic pain (migraine) to healthy controls. Based on these data, a total of 35 subjects are required for power of .8 and p < 0.05. All comparisons were calculated using z-transformed statistical images of the whole brain, a cluster-forming threshold of z>3.1 and p<0.05, and a Gaussian Random Field theory-based approach for multiple comparisons. For these complex data, statistical power is defined as an 80% probability of correctly detecting an active peak for all peaks above the cluster-forming threshold. We note moderate to large effects observed for within and between group studies; thus, a total sample size of 40 ensures were are adequately powered to observe at least moderate (e.g. mean effect size difference of 0.4 or greater) effects. | | | | | | | |
| **10.** | **Benefits.** | | | | | | | |
|  | Describe any potential direct benefit(s) to subjects in this study, if any and the importance of the knowledge that may reasonably be expected to result. Within the description, do not include payment to subjects as a benefit. | | | | | | | |
|  | All participants will be able to receive an evidence-based intervention (ADAPT) either immediately, or after a waitlist period. Preliminary data suggests this intervention has a positive impact on symptoms associated with FAPD and comorbid anxiety. Information obtained from this study will be valuable for refining behavioral interventions for the treatment of youth with chronic pain and comorbid anxiety. | | | | | | | |
| **11.** | **Screening, Recruitment, and Determining Eligibility.** | | | | | | | |
| **11A.** | Describe how subjects will be identified and recruited, including who will perform the recruitment. | | | | | | | |
|  | A research team member will attend GI clinics in person at Helen DeVos Children’s Hospital when possible. When the research team member is not able to attend the clinic (or if the medical visit occurs virtually), the research team member will follow up with the medical team about whether attending patients may meet eligibility criteria for the study. If patient potentially meets criteria, the research team member (who is also an allied healthcare provider at Helen DeVos Children’s Hospital) will approach/contact the patient/family. In addition, patients with FAPD who are referred for behavioral medicine treatment at HDVCH, or who have previously participated in a study of youth with FAPD at HDVCH, may also be introduced to the study by a medical or study team member.  We will recruit males and females to participate in this study. While FAPD is more common in females, we have found that we are easily able to recruit males as well. Based on our prior research projects, we expect that we will recruit a sample of youth with FAPD that is 65% female and 35% male. The PI will check in with the mentorship team quarterly regarding enrollment and gender breakdown in a blinded fashion. If the gender breakdown is skewed (>85% females or >60% males), the PI will problem solve with study mentors to ensure the sample is representative of the population. Recruitment efforts would target candidates from underrepresented gender until the sample is more representative of the FAPD population.  Inclusion Criteria  Participants must ultimately meet all of the inclusion criteria to participate in this study.  • Children (males and females) between 11 and 16 years of age and their parent/primary caregiver will participate in the study.  Child Criteria  • Meets criteria for FAPD based on physician diagnosis of FAPD and ROME IV FAPD criteria (see Description of Evaluations section for additional details).  • Meets criteria for presence of clinically significant moderate anxiety (based on the Generalized Anxiety Disorder-7 [GAD-7] measure with a total cut-off score ≥ 10) or clinician's diagnosis of an anxiety disorder.    Child and Caregiver Criteria  • Youth and caregiver must have sufficient English language ability necessary to complete study measures and protocol.  Exclusion Criteria  All candidates meeting any of the exclusion criteria at screening will be excluded from study participation.  Child Criteria  • Children with a significant medical condition(s) with an identifiable organic cause including those that may include abdominal pain symptoms (e.g., Inflammatory Bowel Diseases).  • Children with a documented developmental delay(s), autism spectrum disorder, a previously diagnosed thought disorder (i.e., psychosis), or bipolar disorder.  • Significant visual, hearing, or speech impairment.  • Organic brain injury.  • Participants who are currently in psychological therapy for pain or anxiety.  • Participants with severe depressive symptoms (T cut-score ≥ 22) on the Patient Health Questionnaire-9 [PHQ-9] or current active suicidal ideation.  • Exclusionary criteria specific to the fMRI component of the study (to be confirmed after consent):  •o Participants with an implant such as a cochlear implant device, a pacemaker or neurostimulator containing electrical circuitry or generating magnetic signals. Participants with any significant ferrous material in their body that could pose the potential for harm in the fMRI environment or cause signal suppression of key regions (i.e. orthodontia).  •o Female participants who report current/suspected pregnancy.  •o Participants with evidence of claustrophobia.  Child and Caregiver Criteria  • Inability or unwillingness of individual or legal guardian/representative to give written informed consent.  Study Enrollment Procedures  • Method for identifying and recruiting candidates for the study. Eligible participants with FAPD will be identified for the study from new or existing participants seen at the outpatient pediatric GI/behavioral medicine clinics, or be recruited from a previous study of youth with FAPD.  • Procedures for documentation of reasons for ineligibility and for non-participation of eligible candidates. A screening log will be maintained with potentially eligible candidates who consented to the study. This log will detail results of screening (e.g., eligibility, ineligibility) and participant progress throughout the study if applicable. Once consented, participant names and ID numbers will be recorded in a secured enrollment log. Of note, reasons for disinterest of non-consented participants will also be recorded without identifiable information.  • Consent procedures. FAPD participants will be introduced to the study in person (or virtually if an in-person option is not available) by a medical staff member and/or study staff member who will explain the study to the patient and the primary caregiver in greater detail. Participants will be assured that their usual medical care will not be affected based upon whether or not they choose to participate. Written consent from the primary caregiver and written assent from the child will be obtained by study staff. All participants will be notified that screening is necessary and study entry is not guaranteed at this point. If the child is not eligible and the family is interested in the child receiving mental health services, contact information for psychology services will be provided.  • Randomization procedure for assigning a participant to an intervention group. Following the baseline visit, patients will then be randomized to either the ADAPT group or a waitlist control group (each six weeks in duration) and will be informed of group assignment within a week of their baseline assessment, and will begin ADAPT/waitlist approximately one week after group assignment (with up to three weeks allowed to begin ADAPT). Gender and age will be used as blocking variables in randomization. | | | | | | | |
|  | *CLICK IRB: Upload the recruitment materials to the Consent Forms and Recruitment Materials SmartForm page, Question 2.* | | | | | | | |
| **11B.** | The study team will obtain for the purpose of screening, recruiting, or determining the eligibility of prospective subjects (please select the appropriate option(s)): | | | | Not Applicable | | | |
|  | Information through oral or written communication with the prospective subject or legally authorized representative. Before the information is obtained for the purpose of screening, recruiting, or determining eligibility, consent:  will be obtained.  will not be obtained. *Please describe screening consent procedures in Question 12.* | | | | | | | |
|  | Identifiable private information or identifiable biospecimens by accessing records or stored identifiable biospecimens. Before the information is obtained for the purpose of screening, recruiting, or determining eligibility, consent:  will be obtained.  will not be obtained. *Please describe screening consent procedures in Question 12.*  *Note: The revised Common Rule permits an exception from informed consent for screening, recruiting, or determining eligibility when certain criteria are met; this exception does not apply to studies subject to the Pre-2018 Common Rule Requirements and/or studies regulated by the U.S. Food and Drug Administration (FDA).* | | | | | | | |
| **11B1.** | Please explain your selection(s). | | | | | | | |
|  | Procedures for documentation of reasons for ineligibility and for non-participation of eligible candidates. Logs will be maintained with eligible candidates approached/contacted for study recruitment. These logs will detail reasons for ineligibility and/or reasons for disinterest in study participation if applicable. For example, if the child/parent declines to participate or the physician has determined the child is medically ineligible, this will be noted so that the family will not be re-approached at a future medical visit.  Once consented, participant names and ID numbers will be recorded in a secured enrollment log. | | | | | | | |
| **12.** | **Consent Process.** | | | | | | | |
| **12A.** | If the study involves adults, consent will be obtained from (select appropriate option(s)): | | | | | Not Applicable | | |
|  | All subjects  Some subjects  No subjects (consent will not be obtained) | | | | | | | |
|  | *CLICK IRB: Upload the consent document, script, etc. (including translations) to the Consent Forms and Recruitment Materials SmartForm page, Question 1.* | | | | | | | |
| **12B.** | If the study involves children, parental permission will be obtained from (select appropriate option(s)): | | | | | Not Applicable | | |
|  | Both parents or guardians (unless one parent is deceased, unknown, incompetent, or not reasonably available, or when only one parent has legal responsibility for the care and custody of the child)  One parent or guardian  Will not be obtained | | | | | | | |
|  | *CLICK IRB: Upload the parental permission forms to the Consent Forms and Recruitment Materials SmartForm page, Question 1.* | | | | | | | |
| **12C.** | If the study involves children, child assent will be obtained from (select appropriate option): | | | | | Not Applicable | | |
|  | All children  Some children  Will not be obtained | | | | | | | |
|  | *CLICK IRB: Upload the child assent form to the Consent Forms and Recruitment Materials SmartForm page, Question 1.* | | | | | | | |
| **12D.** | Describe the consent process, including an explanation of your selection(s) above. If the study involves screening activities, please describe whether consent will be obtained and if consent will not be obtained, explain how the screening data will be used. If only some subjects will provide consent, explain who will or will not provide consent. If only some children will provide assent, explain which children will and will not provide assent. | | | | | | | |
|  | FAPD participants will be introduced to the study in person (or virtually if an in-person option is not available) by a medical staff member and/or study staff member who will explain the study to the patient and the primary caregiver in greater detail. Participants will be assured that their usual medical care will not be affected based upon whether or not they choose to participate. Written consent/permisison from the primary caregiver and written assent from the child will be obtained by study staff. All participants will be notified that screening is necessary and study entry is not guaranteed at this point. If the child is not eligible and the family is interested in the child receiving mental health services, contact information for the psychology service at Spectrum Health will be provided.  A signed assent and consent/permission form will be obtained from each participant and their legal guardian (e.g., person with power of attorney) before completing any study activities. The consent form will describe the purpose of the study, the procedures to be followed, and the risks and benefits of participation. Consent forms will be IRB-approved, and the subject is required to read and review the document or have the document read to him or her. The designee will explain the research study to the subject and answer any questions that may arise. The subject will sign the informed consent document prior to any study-related assessments or procedures. Subjects will be given the opportunity to discuss the study with their surrogates or think about it prior to agreeing to participate. They may withdraw consent at any time throughout the course of the study. A copy of the assent and consent will be given to the legal guardian. | | | | | | | |
| **12E.** | If consent will not be obtained, explain why. Describe why the research could not be practicably carried out if consent was required. If the research involves identifiable private information or identifiable biospecimens, describe why the research could not practicably be carried out without using such information or biospecimens in an identifiable format. | | | | | | | Not Applicable |
|  |  | | | | | | | |
| **12F.** | If your study involves use of a consent form, complete i, ii, and iii. | | | | | | | Not Applicable |
| **12Fi.** | Select the appropriate option(s) below for the documentation of consent.  Will use a written consent document signed by subjects  Will use a short form written consent document signed by subjects  Will not obtain a signed consent document for some subjects  Will not obtain a signed consent document for all subjects | | | | | | | |
| **12Fii.** | Describe when and how the subject will receive a copy of the consent form. | | | | | | | |
|  | Subjects will receive copies of the consent/permission/assent forms at the time of initial enrollment. | | | | | | | |
| **12Fiii.** | If subjects will not be signing the consent document, please explain why. If some subjects will not sign the consent document, explain who will and will not sign the consent. | | | | | | | Not Applicable |
|  |  | | | | | | | |
| **12G.** | If the study involves cognitively impaired adults, explain the process to determine whether a subject is capable of consent, use of any legally authorized representative(s), and any assent process. | | | | | | | Not Applicable |
|  |  | | | | | | | |
|  | *CLICK IRB: Upload any assessment tools to the Supporting Documents SmartForm page.* | | | | | | | |
| **13.** | **Coercion or Undue Influence.** | | | | | | | |
| **13A.** | If some or all of the subjects are likely to be vulnerable to coercion or undue influence, such as children, prisoners, pregnant women, mentally disabled persons, individuals with impaired decision-making capacity, or economically or educationally disadvantaged persons, describe additional safeguards that have been included in the study. | | | | | | | Not Applicable |
|  | All efforts will be made to ensure that youth and their families understand the study and the associated risks and benefits. Participants will have opportunities to voice questions/concerns before formally proving consent/assent. Non-English speakers or those with cognitive delays that would limit understanding of the consenting process will be excluded from participating in the study. It will be made clear that their participation is voluntary and will in no way affect their medical care. | | | | | | | |
| **13B.** | If you or your study team are associated with the subjects (e.g. your students, employees, colleagues, patients), explain the nature of any association and measures taken to protect subjects’ rights, including safeguards against any coercion or undue influence (e.g. pressure a subject might feel to participate based on the association). | | | | | | | Not Applicable |
|  |  | | | | | | | |
| **14.** | **Privacy.** | | | | | | |  |
|  | How will subjects’ privacy be protected? Consider the number of individuals interacting with the subject or subject’s records, location of consent process and study, presence of individuals not associated with the study, sensitivity of the research. | | | | | | | |
|  | To minimize risk to confidentiality, every effort will be made to ensure that research data are kept confidential and stored so that data cannot be accessed by individuals who are not part of the research team. Unique identification numbers will be assigned to participants, and all case report forms will be coded with this number rather than a name. A password-protected master list linking the identification number to participant names will be stored on a secure computer separate from the study data. Access to the master list will be limited to key study personnel. Upon study completion, all study materials and participants’ personal information will be destroyed. Locked filing space within the Secchia Center will be identified and used exclusively for the purposes of this study.  All consent forms, contact information and identifying data will be stored either in a secure location within the Secchia Center or on a secure computer. The subject codebook will be stored separately in a password protected document Regarding the use of online measures and web modules, material development will be conducted in accordance with MSU policies. | | | | | | | |
| **15.** | **Withdrawal of Subjects.** | | | | | | | Not Applicable |
|  | If there are any anticipated circumstances where the researcher will withdraw subjects from the study regardless of the subject’s wishes, describe the circumstances and the procedures when subjects are withdrawn from the study. | | | | | | |  |
|  | The intervention will be discontinued if a participant experiences an increase in distress and pain symptoms throughout the intervention, or if the interventionist decides that a participant’s presentation warrants more targeted care outside of what the intervention offers. The intervention will be discontinued for all participants in the event of a study closure by the institute. | | | | | | | |
| **16.** | **Monitoring Plan to Assess Data to Ensure Safety of Subjects.** | | | | | | |  |
| **16A.** | Is there a monitoring plan to periodically assess the data to ensure the safety of subjects or to ensure negative outcomes do not occur? | | | | | | | No  Yes |
|  | Explain your answer. If you answered Yes, describe the monitoring plan. | | | | | | |  |
|  | The research team will also report any significant study-related or unanticipated adverse events to the Institutional Review Board and to the study sponsor based upon institutional and sponsor guidelines. In addition, an Independent Monitoring Committee (IMC) will be formed to assess the safety and study-related concerns. Any reportable events that occur (which includes the occurrence of any safety issues related to the scanning procedures or to a breakdown of confidentiality) will be reported to the IRB immediately. We will also regularly communicate with NCCIH regarding safety monitoring practices. | | | | | | | |
|  | *CLICK IRB: Upload any data safety monitoring plans to the Supporting Documents SmartForm pages.* | | | | | | | |
| **16B.** | If there is a data safety monitoring committee or board, describe the composition and frequency of meetings. | | | | | | | Not Applicable |
|  | The IMC will meet annually and will communicate quarterly or more frequently as needed outside of the annual meetings via email. | | | | | | | |
| **17.** | **Results and Data Sharing.** | | | | | | |  |
| **17A.** | Could this research generate any results that could be clinically relevant, including individual research results, or general, or aggregate research findings? | | | | | | | |
|  | No  Yes, clinically relevant individual research results  Yes, clinically relevant general or aggregate research findings | | | | | | | |
| **17A1.** | If yes, explain what clinically relevant research results will be generated, whether they will be disclosed to subjects or others (e.g. subject’s primary care physician), and if so, under what conditions. Address individual research results and/or general or aggregate research findings, as appropriate. *This also needs to be explained in the consent document.* | | | | | | | |
|  | Preliminary data already suggests the ADAPT intervention may have a positive impact on symptoms associated with FAPD and comorbid anxiety. The proposed research aims to understand neural mechanisms of effect of the intervention. | | | | | | | |
| **17B.** | For other research results, select all that apply: | | | Not Applicable | | | | |
|  | Overall study results will be shared directly with subjects  Individual results or incidental findings of individual subjects will be shared with subjects or others  Data will be submitted to a repository or database as part of data sharing agreement (e.g. genomic data sharing) | | | | | | | |
| **17B1.** | Explain your selection(s), including how the data or results will be shared and with who (e.g. subject’s primary care physician, data repository). | | | | | | | |
|  | Neuroimaging data will be shared in a data repository (TBD) upon completion of the study. | | | | | | | |
| **18.** | **Local Context and Multi-Site Study.** | | | | | | | |
| **18A.** | Describe the locations of where the study team will obtain information or biospecimens through intervention or interaction with the subject or obtain the subjects’ private identifiable information. | | | | | | | |
|  | MSU Department of Family Medicine (Secchia Center) and the Helen DeVos Children's Hospital. | | | | | | | |
| **18B.** | If the study will engage employees or agents of non-MSU organizations (e.g. performance sites), explain how the employees or agents will be engaged (e.g. will they perform research procedures, will they obtain informed consent from subjects). | | | | | | | Not Applicable |
|  | The study psychologist (Dr. Brittany Barber Garcia) will provide ADAPT to patients and maintain the blind. Dr. Ismaeel Hashemi will help identify potential eligibile participants until 05/19/23, at which point Dr. Ryan Cox will assume the position. Study team members from Spectrum Health may also obtain consent/assent from patients for the study.  In addition, Taylor Abounader of Wright State University will help deliver the ADAPT intervention. | | | | | | | |
| **18C.** | If the study involves multiple performance sites, describe the methods for communicating with engaged sites related to the protection of human subjects (e.g. any potential unanticipated problems that may involve risks to subjects others). | | | | | | | Not Applicable |
|  |  | | | | | | | |
| **18D.** | If there are any cultural or local contexts or requirements that may impact the protection of human subjects or present additional risks to subjects that have not otherwise been described, please describe. If research is conducted outside the state of Michigan, this could include additional state or international requirements or laws. | | | | | | | Not Applicable |
|  |  | | | | | | | |
| **18E.** | If translations to a language other than English will be provided to subjects, describe the translation process. | | | | | | | Not Applicable |
|  |  | | | | | | | |
|  | *CLICK IRB: Upload translated documents to the appropriate SmartForm page(s).* | | | | | | | |
| **19.** | **Resources and Financial Compensation and Costs.** | | | | | | | |
| **19A.** | If someone will receive a payment for recruiting the subjects, explain the amount of payment, who pays it, who receives it, and why they are being paid. | | | | | | | Not Applicable |
|  |  | | | | | | | |
| **19B.** | If subjects will incur additional financial costs as a result of their participation in this study, explain the additional costs. | | | | | | | Not Applicable |
|  |  | | | | | | | |
| **19C.** | Describe any resources not otherwise described elsewhere in the submission (e.g. internal funding) for the protection of human subjects. | | | | | | | Not Applicable |
|  |  | | | | | | | |
|  | *CLICK IRB: Upload any funding materials not accessible in Kuali Coeus in the Supporting Documents SmartForm page*. | | | | | | | |
| **19D.** | If subject’s biospecimens (even if identifiers are removed) may be used for commercial profit, describe whether the subject will or will not share in the commercial profit. *This also needs to be explained in the consent document.* | | | | | | Not Applicable | |
|  |  | | | | | | | |
| **20.** | **Information and/or Biospecimen(s) Management and Confidentiality.** | | | | | | | |
| **20A.** | Select the appropriate option: | | | | | | | |
|  | Identifying or coded information will not be stored with the information and/or biospecimen(s)  Identifying or coded information will be stored with the information and/or biospecimen(s) | | | | | | | |
| **20B.** | Please explain your selection. If you are storing identifying or coded information with the information and/or biospecimen(s), explain why identifiable or coded information and/or biospecimen(s) needs to be maintained and how long it will be necessary to maintain it. | | | | | | | |
|  | Identifiable information may be stored on a secure REDCap database which will also contain the participant's unique idenitfication number. However, this identifiable information will be stripped for data analysis. | | | | | | | |
| **20C.** | Describe the procedures and safeguards you will use to secure the information and/or biospecimen(s), including during transport of information and/or biospecimen(s). | | | | | | | |
|  | To ensure confidentiality, identification numbers will be used on data collection forms in lieu of names. Regarding the use of online measures and web modules, material development will be conducted in accordance with MSU policies. Hardware for this study will be provided and maintained by MSU, which maintains a secure web server for supporting projects that potentially contain protected health information (PHI) and are subsequently subject to compliance with federal and state regulations regarding data of this type. | | | | | | | |
| **21.** | **Drug and/or Device Storage, Handling, and Administration.** | | | | | | | Not Applicable |
|  | Describe the procedure and plan for storage, handling, and administration of the drug and/or device so that they will be used only on enrolled subjects and be used only by authorized study personnel. | | | | | | | |
|  |  | | | | | | | |
| **22.** | **Future Research.** | | | | | | |  |
|  | If the research involves the collection of identifiable private information or identifiable biospecimens, select the appropriate option: | | | | | | | Not Applicable |
|  | The subject’s information or biospecimens, even if identifiers are removed, could be used for future research studies or distributed to another investigator for future research studies  The subject’s information or biospecimens, even if identifiers are removed, will NOT be used or distributed for future research studies | | | | | | | |
|  | *Please be sure to carefully consider the appropriate option, as this needs to be explained in the informed consent and can limit what is done or used for future research.* | | | | | | | |
| **23.** | **MSU Additional Information.** | | | | | | | Not Applicable |
|  | Identify if your study involves any of the following: (check all that apply) | | | | | | | |
|  | Use of human stem cells  Research with biospecimens will (if known) or might include whole genome sequencing (i.e., sequencing of a human germline or somatic specimen with the intent to generate the genome or exome sequence of that specimen). *If so, this needs to be explained in the consent document.* | | | | | | | |
|  |  | | | | | | | |
|  | **Other Click IRB Document Uploads As Appropriate**  **(Applicable to Expedited or Full Board Studies)** | | | | | | | |
|  | - *Upload list of external study team members (non-MSU individuals) to the Study Team Members SmartForm page, Question 2.* - *Upload other institution(s) approval letter(s), if submitted to other IRB(s) or ethics committees, to the Supporting Documents SmartForm page.* - *Upload FDA communications, package inserts, FDA form 1572, or other information related to drugs or devices to the appropriate Drug or Device SmartForm pages.* - *Upload the HRP-540 - Template - ICH-GCP - For Investigator to the MSU Additional Study Information SmartForm page.* - *Upload HRP-541 - Template - Involvement of Prisoners in a Research Project to the MSU Additional Study Information SmartForm page.* - *Upload the investigator brochure to the Supporting Documents SmartForm page* - *Upload the MRI Screening Form – Women to the Supporting Documents SmartForm page.* - *Upload the translation of instrument(s) provided to non-English speaking subjects to the Supporting Documents SmartForm page.* - *Upload the curriculum vitae(s) when research is more than minimum risk to the Supporting Documents SmartForm page.* - *Upload case report forms to the Supporting Documents SmartForm page.* - *Upload the Non-MSU Employee Conflict of Interest Disclosure Form to the Supporting Documents SmartForm page.* - *Upload any other pertinent documents related to the proposed research study to the Supporting Documents SmartForm page* | | | | | | | |
